# The uncharted territory of host-pathogen interaction in tuberculosis

**DOI:** 10.3389/fimmu.2024.1339467

**Published:** 2024-01-19

**Authors:** Antara Ghoshal, Akanksha Verma, Ashima Bhaskar, Ved Prakash Dwivedi

**Affiliations:** Immunobiology Group, International Centre for Genetic Engineering and Biotechnology, New Delhi, India

**Keywords:** *Mycobacterium tuberculosis*, immunometabolism, ER stress, microbiome, exosomes, cell-free DNA, biomarkers

## Abstract

*Mycobacterium tuberculosis* (*M.tb*) effectively manipulates the host processes to establish the deadly respiratory disease, Tuberculosis (TB). *M.tb* has developed key mechanisms to disrupt the host cell health to combat immune responses and replicate efficaciously. *M.tb* antigens such as ESAT-6, 19kDa lipoprotein, Hip1, and Hsp70 destroy the integrity of cell organelles (Mitochondria, Endoplasmic Reticulum, Nucleus, Phagosomes) or delay innate/adaptive cell responses. This is followed by the induction of cellular stress responses in the host. Such cells can either undergo various cell death processes such as apoptosis or necrosis, or mount effective immune responses to clear the invading pathogen. Further, to combat the infection progression, the host secretes extracellular vesicles such as exosomes to initiate immune signaling. The exosomes can contain *M.tb* as well as host cell-derived peptides that can act as a double-edged sword in the immune signaling event. The host-symbiont microbiota produces various metabolites that are beneficial for maintaining healthy tissue microenvironment. In juxtaposition to the above-mentioned mechanisms, *M.tb* dysregulates the gut and respiratory microbiome to support its replication and dissemination process. The above-mentioned interconnected host cellular processes of Immunometabolism, Cellular stress, Host Microbiome, and Extracellular vesicles are less explored in the realm of exploration of novel Host-directed therapies for TB. Therefore, this review highlights the intertwined host cellular processes to control *M.tb* survival and showcases the important factors that can be targeted for designing efficacious therapy.

## 1 Introduction

**Figure 1:**
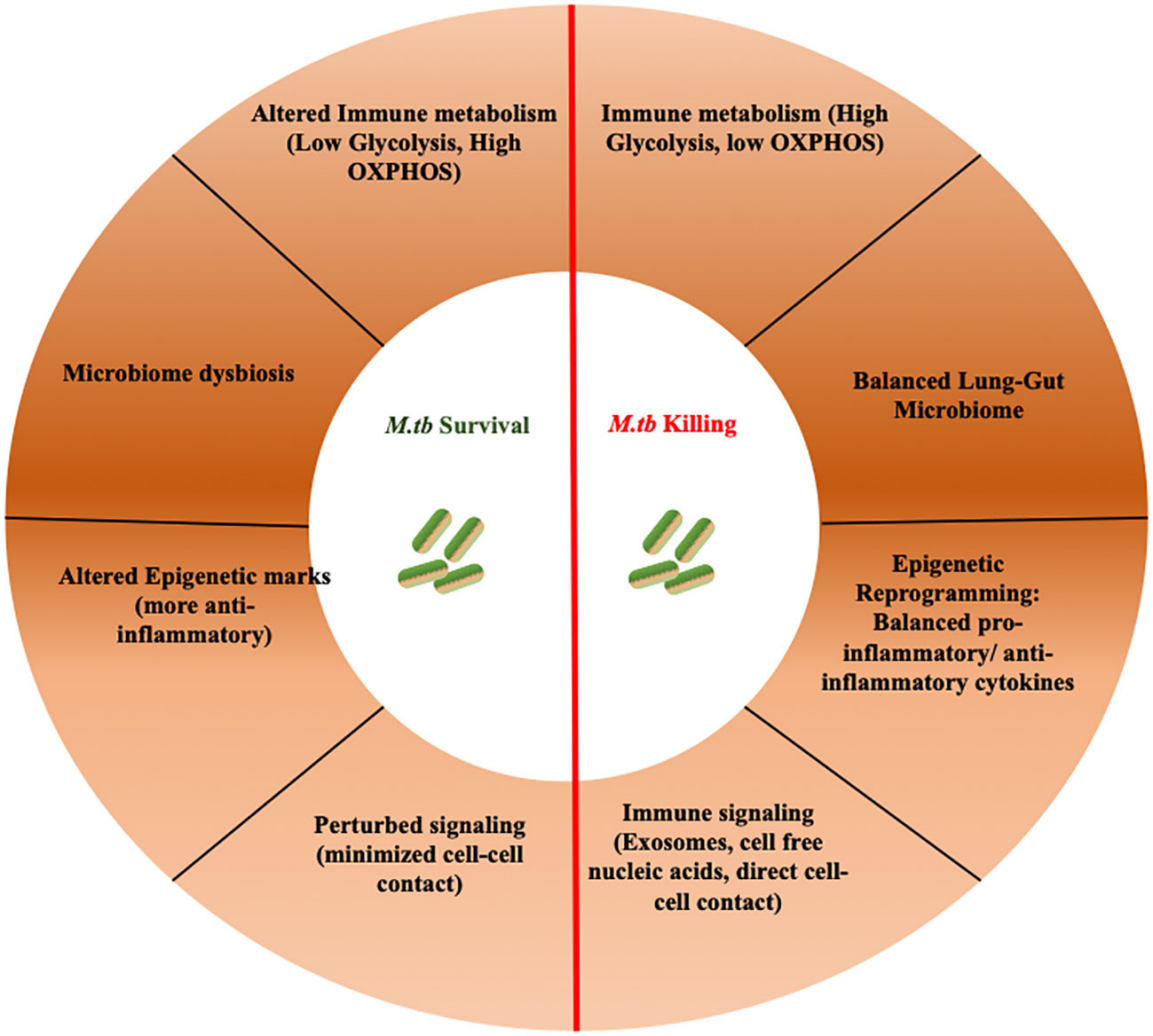
Over-view of various integrated host processes that governs TB progression. TB progression is influenced by an interplay of integrated host processes. These intricate mechanisms govern the pathogenesis of TB, balancing disease clearance and progression.

*Mycobacterium tuberculosis* (*M.tb*), the causative bacterium of the deadly disease Tuberculosis (TB) stands as one of humanity’s most enduring pathogens (1). The extensive co-evolutionary history has equipped *M.tb* with formidable arsenals to counteract host immune responses (2). The interplay between the host and pathogen represents a highly intricate and ever-evolving phenomenon that is pivotal in determining disease progression or the successful clearance of the invading pathogen (3). The dynamic relationship between *M.tb* and the host immune system operates at multiple levels, encompassing various cellular processes, such as immune signaling, metabolic responses, the management of various cellular stresses (including those affecting the endoplasmic reticulum, mitochondria, and DNA integrity), host-microbiome balance, the release of extracellular components like exosomes and cell-free nucleic acids, and many other regulatory factors (4, 5) (**Figure 1**). For instance, *M.tb* deploys multiple factors such as Hip1, ESAT-6, etc, to delay T-cell responses by targeting immune metabolism (6). The immune cells, in turn, maintain high ATP levels to synthesize and secrete pro-inflammatory cytokines to limit *M.tb* infection (7).

The source for the functioning of host immune cells depends on several metabolic pathways. There is a reciprocal relationship between the host and *M.*tb in terms of their metabolism. The presence of the host-microbiome adds complexity to the host immune system functioning (8). The microbiome-derived metabolic intermediates are involved in intricating host immune signaling pathways. Studies using the murine model have illustrated the differential expansion of T cell subsets in the presence of varying gut symbionts. For instance, the Th1 response dominates in mice colonized with *Bacteroides fragilis* (9) while the Th2 response is predominant in mice colonized with Clostridial strains (10). Antibiotic treatment such as Isoniazid/Pyrazinamide has been observed to alter the gut microbiome in TB murine model. The altered gut microbiome changes the metabolites in peripheral circulation which in turn has been found to alter the metabolism of alveolar macrophages. This results in defective microbicidal activity and enhanced susceptibility of macrophages to *M.tb* (11, 12).

Apart from impacting the host immune system, the metabolites can also have a direct anti-mycobacterial activity (13). For instance, Indole-3 propionic acid (IPA) generated from tryptophan (trp) metabolism by gut symbiont *Clostridium sporogenes* was found to showcase anti-mycobacterial properties in a murine model of TB (14). Thus the metabolites derived from the gut symbionts impact *M.tb* survival/clearance either through direct anti-mycobacterial activity or indirectly through the activation of host immune responses.

The translocation of metabolites and folded/unfolded proteins from infected cells initiates signaling processes for the immune cells. Host and *M.tb* derived proteins are transported through the exosomes. The contents of exosomes act as a double-edged sword. Exosomes have been found to promote protective Th1mediated immunity during TB in murine models (15). Despite positive immune activation by exosomes, few studies have elucidated the negative immune regulation during TB. In the later stages of macrophage infection by *M.tb*, a notable decrease in macrophage activation followed by upregulation of apoptotic and autophagic pathways has been studied (16, 17).

Understanding and decoding these complex cellular and microbial events will help us understand TB in a more effective way. It is noteworthy to realize that the host cellular processes mentioned above are intertwined and maintain the symbiotic relationship between the host and *M.tb.* In the contemporary scientific landscape, researchers are delving extensively into characterizing host-*M.tb* interactions that will aid in decoding *M.tb* pathogenesis (1, 18). This endeavour is essential for developing more effective immunotherapies that can enhance protection against active TB (ATB), latent TB (LTBI), and reactivation of the infection.

This review provides a concise overview of the lesser-explored host processes- Immunometabolism, Endoplasmic (ER) stress, Host microbiome, and Extracellular vesicles. These processes are few amongst the countless host processes such as transcriptional and translational reprograming, innate/adaptive cells activation, inflammatory mediators signaling, cellular metabolism, granuloma formation, cell death and survival processes etc, which are involved in the dynamic interaction between the host and *M.tb*. These crucial interactions offer potential targets for the development of innovative Host Directed Therapies aimed at combating TB and warding off the emergence of antibiotic-resistant strains.

## 2 Canonical pathogenesis of *M.tb*

*M.tb* interacts with numerous biomolecules and different cell types to establish the infection successfully. Upon aerosol inhalation, *M.tb* interacts with respiratory epithelial cells and persists for nearly 48 hours without replicating (19). The general notion in the early stage of *M.tb* infection implies that *M.tb* released from dying, necrotic cells interact higher with alveolar epithelial cells, irrespective of replication (20).These epithelial cells are well known to modulate the local immune microenvironment against *M.tb* as they secrete pro-inflammatory molecules that drive the myeloid cell activation (19). This is followed by the entry of *M.tb* through M cells to the underlying lymphoid layer and predominantly infecting the alveolar macrophages through pattern recognition receptors (PRRs). The PRRs recognize various pathogen-associated molecular patterns (PAMPs) present or secreted by *M.tb* Such molecules are mycolic acids, lipoarabinomannan (LAM), and secreted proteins such as ESAT-6, facilitating the activation of the MyD88 signaling pathway. This, in turn, induces an inflammatory response to control *M.tb* infection. Some studies reported that *M.tb* induces Irg1 expression via MyD88 and STING pathway, eventually controlling *M.tb* infection (21). It has been demonstrated that *M.tb* lipids such as phthiocerol dimycocerosate (PDIM) facilitate the fusion of *M.tb* with the membrane of macrophages and ensure successful entrance to the cell through the endocytic pathway (22). Within these macrophages, *M.tb* may either be degraded by lysosomal acidification or autophagy, or it may surpass these barriers to infect other immune cells such as dendritic cells, neutrophils, or myeloid cells (23). These infected tissue-resident alveolar macrophages initiate signaling cascades which is essential to synthesize anti-microbial peptides, pro-inflammatory cytokines, and prostaglandins to effectively clear *M.tb.* Macrophage pro-inflammatory phenotype is obtained by shifting predominantly to glycolysis, initiating transcriptional programs that supports acquisition of an anti-bacterial state (24). *M.tb* has adopted various mechanisms to subvert the lysosomal degradation within the macrophages. The cell wall organization of *M.tb*, especially mannose-capped lipoarabinomannan (manLAM) sanctions the survival within the phagosomes of the macrophages by preventing phosphorylation of phosphatidylinositol-3-phosphate (PI3P) and further prevents maturation of early phagosomes to late phagosomes (25, 26). Nonetheless, *M.tb* utilizes its type VII secretion system and secretes ESAT-6 to exit out of these macrophages and infect neutrophils and dendritic cells in the lung interstitium (27).

Recent pieces of evidence show that TB patients have a higher number of neutrophils which is associated with the failure of TB regimen, i.e., anti-tubercular therapy, and show higher lung pathology (28). Within neutrophils, *M.tb* replicates and is subjected to degradation by an arsenal of proteolytic enzymes such as elastase, proteinase 3, cathepsin G, and Reactive oxygen species (ROS) (29). SLAMF1 is one of the co-stimulatory proteins that governs autophagy in neutrophils, and projects *M.tb* degradation. However, SLAMF1 levels are downregulated in TB patients when compared to healthy controls, and thus unable to control *M.tb* progression (28). Neutrophils govern the adaptive immune response by directing dendritic cells (DCs) to present antigens to T cells (30). There are two classes of thoughts for the role of neutrophils in TB (31). Firstly, neutrophils are known to control the early stage of infection during pulmonary TB (32), whereas another study suggests that neutrophils are permissive to *M.tb* and help in disease progression during the granulomatous stage of infection, and cause tissue destruction (33). In later stages of infection, neutrophils expedite the growth of the bacteria and facilitate the dissemination of *M.tb* (33). It has been shown that neutrophils interact with T cells and macrophages within the granuloma and secrete immunosuppressive cytokines that downregulates T cell responses (34). The detailed role of neutrophils during different stages of *M.tb* infection is yet to be elucidated.

It is conventionally known that innate immune cells provide the first line of defense against *M.tb* and commands adaptive immune cells to contain the bacteria within the hypoxic granulomas. This bridging is primarily done by antigen-presenting cells, mainly dendritic cells that are least phagocytic in nature. They phagocytose *M.tb* and degrade it to derive *M.tb* antigens. The infected DCs then migrate to the distal lymph nodes in response to CCL19/21 gradients to induce effective cell-mediated immunity (35). Once, enough antigens are generated, DCs downregulate their phagocytic activity and divert their energy status in antigen processing and presentation to T cells. However, accumulating shreds of evidence suggest that *M.tb* has employed various mechanisms to delay the ability of DCs to present antigens to T cells (26). Certain *M.tb* antigens such as serine hydrolase Hip1, ESAT-6, and hsp70 impair the maturation and migration of DCs and thereby prevent T cell proliferation (36–38). Another *M.tb* antigen 19kDa lipoprotein is known to downregulate the expression of MHC class II and HLA-DM on DCs, thereby delaying the T cell priming (39, 40). This poses barrier in restricting the infection, and hence *M.tb* disseminates to other organs such as the central nervous system, kidney, liver, pancreas, etc (41). As DCs present *M.tb* antigens to naïve CD4^+^ T cells via MHC II, the CD4^+^ T cells are activated and subsequently differentiated into Th1 type to secrete IFN-γ, TNF-α, and IL-2. These activated CD4^+^ T cells are crucial in containing the *M.tb* within granuloma, and further activating CD8^+^ T cells to enable its cytotoxic effector functions. CD4^+^ T cells act synergistically with CD8^+^ T cells to prevent the growth of *M.tb* (42). However, chronic exposure to *M.tb* antigens leads to T cell exhaustion in which T cells are functionally impaired. Such T cells express TIM-3 along with other co-inhibitory molecules such as PD-1, LAG-3, CTLA-4 which results in lower production of IL-2, IFN-γ, and other pro-inflammatory cytokines (43). It is also reported that CD4^+^ T cells are necessary to prevent CD8^+^ T cell exhaustion (42). Conversely, a recent report suggests that patients with Tuberculous Pleural Effusion (TPE) have higher numbers of inflammatory granzyme-secreting CD8^+^ T cells in the pleural space, possibly involved in disease progression (44). Single-cell RNA sequencing and TCR sequencing have revealed different subsets of T cells in different compartments of infected lungs in TPE patients (44). This is necessary to realize that the migration of activated T cells to the site of infection is primarily based on the expression of chemokines and their cognate receptors. CCL5, CCL19, and CCL21 are the only known chemokines that facilitate the migration of T cells from distal lymph nodes to the infected lungs (45, 46).

The collective efforts and responses of immune cells to defeat the so-called ‘Trojan horse’ are indispensable. Contrarywise, *M.tb* has evolved its arsenal of immune-escaping and immune-suppressive mechanisms to conquer the complex processes of the host (17). It is important to highlight that both host and *M.tb* are genetically complex and thus harbor various other pathways of interactions, which are yet to be investigated.

## 3 *M.tb* impacts various host processes impacting immune system- conferring disease progression/resistance

Various host processes collectively regulate disease pathogenesis, which are conquered by *M.tb.* Such processes involve DNA replication, transcriptional reprogramming, translation, cell death pathways, cell survival, metabolism, cell transport process, stress events such as ER stress, mitochondrion stress, etc. These processes integrate to control *M.tb* infection, and prevent tissue pathology. In this review, we aim to focus on Immunometabolism, ER stress, Microbiome and Exosome mediated immune signaling that modulates the host-*M.tb* interaction dynamics.

### 3.1 Immunometabolism

The process of mounting an antimicrobial response and inducing effector functions is a thermodynamically and energetically expensive process in immune cells (47). The cells employ bioenergetics to meet the energy demand according to their status. These needs are met by metabolic plasticity that serves to be crucial for macrophages and T cells to synthesize antimicrobial molecules and prompt effector functions (48). Conventionally, the integrated processes involving glycolysis, tricarboxylic acid cycle (TCA), oxidative phosphorylation (OXPHOS), and fatty acid metabolism supply the energy demands universally in all cell types at their quiescent stage (49, 50). However, metabolic plasticity is central in immune cells as these cells execute highly energy-intensive functions (51). This includes increased synthesis of antimicrobial peptides, pro-inflammatory and anti-inflammatory cytokines, chemokines and their cognate receptors, antigen processing and presentation, synthesis of immunoglobulins and exhibit cytolytic functions (52). It is crucial to highlight that immunometabolism is influenced by the stage of *M.tb* infection such as active state, re-infection and latent TB (granuloma) (53, 54). In this section, we highlighted the alterations between metabolic processes and host immune cells upon different stages of *M.tb* infection.

#### 3.1.1 Macrophages

During active state, upon *M.tb* phagocytosis through toll-like receptors (TLRs), especially TLR2/4, the alveolar macrophages become activated in MyD88 dependent manner which leads to acquiring a pro-inflammatory ‘M1’ phenotype (55). ‘M1’ macrophages require a massive number of ATPs to synthesize pro-inflammatory cytokines such as IL-1β and antimicrobial peptides, therefore these macrophages reprogram the metabolic processes and shift to glycolysis to meet their energy requirements (56). Laura et al., have shown that *M.tb* induces a metabolic shift towards aerobic glycolysis and perturbs OXPHOS in alveolar macrophages (57). This results in the accumulation of TCA cycle products such as succinate that induces the expression of hypoxia-inducible factor 1α (HIF-1α) (53). HIF-1α is the master regulator of the glycolysis pathway that regulates the expression of glycolytic enzymes which further acts in a positive feedback manner to increase the glycolysis (58). Metabolic plasticity in macrophages has been extensively reviewed elsewhere (53, 59). It has been broadly studied that *M.tb* induces ‘Warburg effect’ by increasing the expression of glucose receptors that ultimately leads to enhanced uptake of glucose within the infected macrophages (60). This is accompanied by the increase in end- product of glycolysis, i.e., lactate secretion in extracellular spaces. It has been demonstrated that lactate secreted from the infected cells drive the negative regulation of glycolysis in the resting macrophages (61). The decrease in glycolysis is balanced by the increase in OXPHOS, thereby lactate act as immunomodulator by inhibiting the production of IL-1β and TNF. However, it is interesting to note that lactate rewires the metabolic responses in resting macrophages so that subsequent infection with *M.tb* is diminished (61). Conversely, a recent study suggests that enhanced glucose uptake or during Diabetes Mellitus in TB patients (TB-DM), the increased glucose levels negatively regulate HIF-1α, and thus mediates the survival of *M.tb* in the infected macrophages (62). Another study demonstrated that alveolar macrophages offer nutrient rich and permissive environment for *M.tb* and thus enables higher bacterial replication when compared with interstitial macrophages Further sequencing analysis revealed that alveolar macrophages have higher fatty acid oxidation that allows release of fatty acids and cholesterol available for uptake by *M.tb* (63). Furthermore, *M.tb* maintains low replicating stage, and sustain in extracellular compartment within the granuloma. This is likely due to low amounts for fatty acids and cholesterol, which are necessary for its replication (64).

Nonetheless, *M.tb* has evolved key mechanisms to drive the transition from M1 to M2 phenotype by ravaging mitochondrial integrity. *M.tb* secretes ESAT-6 that damages mitochondrion upon mTOR inhibition, destroys mitochondrial membrane and disrupts the membrane potential (65). This in turn interrupts the contacts between mitochondrion and phagolysosome, which is thought to be necessary for *M.tb k*illing (10.1016/j.micinf.2015.06.003). Another defensive strategy employed by *M.tb* is delayed apoptosis and enhanced upregulation of OXPHOS in *M.tb* infected macrophages. Rv1813c is an effector *M.tb* protein that when overexpressed in macrophages, is deposited in mitochondrial matrix and prevents the release of cytochrome c (6). However, *M.tb* secretes various potent antigenic proteins that collectively promotes *M.tb* survival, but their collective role in altering macrophage metabolism is yet to be studied. Also, this has been known for ages that during latent TB, *M.tb* secretes ESAT-6 in granuloma during latent TB, and simultaneously, macrophages exhibit anti-inflammatory properties (66). However, least efforts have been made to visualize what happens at metabolic level that do not allow the macrophage cell death, even with loss of mitochondrial integrity. Metabolomic analysis of *M.tb* infected macrophages revealed higher levels of NAD^+^, glutathione and creatinine, and it has been reported that these metabolites collectively regulate anti-mycobacterial activity of macrophages as compared to uninfected macrophages (56). Additionally, *M.tb* upregulates glutathione metabolism in macrophages resulting in higher levels of glutamine, that supports M2 polarization (67). Recent studies have classified ratios of amino acids in plasma to whole blood as biomarkers with 84% sensitivity in detecting active TB patients. Amino acids such as leucine, asparagine, ornithine, arginine, tyrosine and serine are present in higher ratios in TB patients when compared to cured TB patients and healthy controls (68). These are probably secreted in the blood and are taken up by macrophages for Nitric oxide (NO) production. Conversely, one of the interesting studies suggested that administration of L-tyrosine curbs mycobacterial survival in TB granulomas, possible due to increased production of ROS (69). However, amino acid metabolism in host macrophages is yet to be elucidated with respect to TB progression.

#### 3.1.2 T cells

Innate immune cells such as macrophages and DCs bridge the antigen encounter with naïve adaptive immune cells through antigen presentation. *M.tb* infected M1 macrophages secrete certain cytokines such as IL-6, IL-10, and IL-12 in the local environment where naïve T cells sense these cytokines, and enhance their glycolysis to prompt effector functions (70). Together with this, T cells need to proliferate and expand clonally which necessitates the rapid synthesis of proteins, lipids and DNA. These phenotypes are acquired by rapid nutrient uptake such as glucose, and other metabolites, and calcium mobilization. In accordance to T cell effector functions, ketolysis is an alternative to glycolytic pathway that drives CD8^+^ T cell cytolytic and cytokines functions such as IFN-γ and TNF-α (71). It is relevant to investigate ketolysis in TB progression and to delineate the possible protective mechanisms against TB.

In general, TCR interaction with co-stimulatory molecules such as CD80/86 induces mitochondrial biogenesis, that also enhances the upregulation of AMP kinase, and further supplements ATPs for rapid T cell proliferation and effector functions (72). A remarkable study demonstrated that mitochondrial matrix protein, Cyclophilin D (CypD) controls *M.tb* infection by controlling T cell responses, and maintains disease tolerance. In CypD deficient *M.tb* infected mice, there is higher infiltration of T cells in lungs, which leads to lung pathology. The study has established that CypD tightly controls aerobic glycolysis in T cells in ROS dependent manner that is known for inducing effector functions such as production of IFN-γ, TNF-α and IL2 (73). As discussed by Koeken et al., T cells and other myeloid cells are evolving in conjunction with *M.tb* to promote disease tolerance that eventually leads to lower immunopathology (74).

Nonetheless, chronic exposure of *M.tb* results in T cell dysfunction, primarily, CD8^+^ T cells. *M.tb* exposure stems metabolic reprogramming in T cells which subsequently ensues the expression of exhaustion markers such as PD-1, CTLA-4 (75). Through transcriptomic and experimental analysis, these observations are finely correlated with reduced glucose uptake by *M.tb* specific CD8^+^ T cells. The series of bioenergetics further demonstrated that chronic exposure of CD8^+^ T cells dampens mitochondrial health, declining OXPHOS, and reduced production of cytokines, which marks T cell exhaustion (75). However, CD4^+^ T cells are known to prevent CD8^+^ T cell exhaustion during *M.tb* infection by an unknown mechanism to produce more IFN-γ, TNF-α, granzymes and perforins (42). Based on these evidences, this could be speculated that CD4^+^ T cells modulate CD8^+^ T cells by preventing mitochondrial dysfunction, and more pronounced aerobic glycolysis. Nonetheless, these aforementioned speculations need experimental validations and analysis. Conventional to the metabolism in all immune cells, HIF1α is known in CD4^+^ T cells to upregulate glycolysis to produce ATP rapidly. This is paramount for release of cytokines such as IFN-γ that drives macrophage activation. Nevertheless, using conditional knockout mechanisms, Liu et al., have shown that deletion of negative regulator of HIF-1α, VHL, dampens the host’s ability to control *M.tb* infection, and hence lower accumulation of *M.tb* specific T cells in the lungs of these mice. This is explained by the fact that VHL is vital for stabilizing the appropriate extent of HIF1α in controlling CD4^+^ T cell response against *M.tb*. This further accompanies to prevent CD4^+^ T cell exhaustion during *M.tb* infection. Altogether, VHL is required for T cell activation, DNA replication and proliferation (76).

The above- mentioned reports are well described in the context of effector functions of T cells. Conversely, minimal efforts are put into identifying the metabolic reprogramming in stemming the memory responses of T cells, which are crucial for the basis of any therapeutic development. Our group has previously shown that *M.tb* induces higher expression of specific histone deacetylase (HDACs), such as Sirtuin 2 in CD4^+^ T cells that deacetylates NF-ĸB at lysine 310 which impairs macrophage- CD4^+^ T cells interactions to restrict *M.tb* growth (77). Recently, the study from our group has discovered that pharmacological inhibition of Sirtuin 2 by AGK2 induces glycolysis by modulating Wnt-β-catenin pathway, that subsequently enhances T-cell stem cell like memory (CD4^+^ T_scm_) responses during *M.tb* infection. This is further complemented by adjunct therapy with BCG as well as DOTS therapy, that results in higher T_scm_ responses that further give rise to different memory pools and effector T cells (77, 78). Similarly, whole proteomics analysis of Purified protein derivative (PPD^+)^ healthy individuals when treated with an immunomodulator, Berberine, showed upregulation of glycolytic pathway along with NOTCH3, that may possibly play role in prompting protective T cell effector function against TB (79).

In conclusion, it is imperative to note that delving into immunometabolism of the host during *M.tb* infection will help researchers refine the vaccine development. By incorporating insights into the metabolic requirements of immune cells during M.*tb* infection, researchers can design vaccines that enhance the overall efficacy of the immune response, potentially leading to more successful prevention and treatment strategies for TB.

### 3.2 Endoplasmic reticulum stress

Apart from modulating immunometabolism, *M.tb* induces ER stress as its survival strategy, and hijack the host immune responses (80). ER stress is caused due to imbalance between protein synthesis and protein folding. Consequently, unfolded proteins accumulate in the cell and induce ‘Unfolded Protein Response’ (UPR) (81). This UPR/ER stress is caused by *M.tb* during ATB and LTBI for retrieving nutrients as well as evading immune responses. Siemons et al., reported for the first time in 2010 that *M.tb* infection induces ER stress-related genes in granulomas in humans and mice. Immuno-histochemistry demonstrated that the markers of ER stress such as CHOP, eIF2α, Ire1α, and ATF3 have higher expression in the *M.tb* infected macrophages within human and mice granulomas (82). Thereafter, there has been a surge in identifying *M.tb* proteins that induce ER stress and the significance of ER stress during *M.tb* pathogenesis. There are couple of *M.tb* proteins identified till date that induce ER stress for survival, dissemination and nutrient uptake. Through over-expression studies, Choi et al., reported that ESAT-6 induces ROS in A549 epithelial cells by increasing intracellular Ca^2+^ concentration, that subsequently induces ER stress (83). Moreover, ER stress induction subsequently triggers apoptosis of the infected cells through activation of Caspase-12 that lies near ER compartment, hence modulates intracellular survival of *M.tb* (84). This study has demonstrated that initially higher expression of ER chaperones, BiP is essential for host cell survival, whereas later during *M.tb* infection, *M.tb* induces higher expression of CHOP pathway, that subsequently activates Caspase-12 to induce apoptosis (84). Conventionally, apoptotic bodies carrying *M.tb* undergo efferocytosis, and hence helps in reduction in *M.tb* burden. However, researchers delved deeper into identifying what provokes ER stress-induced apoptosis in *M.tb* infected cells. Further, they observed that another ER chaperone, Calreticulin is upregulated upon *M.tb* infection. Calreticulin forms complex with CXCR1 and TNFR and induces apoptosis, thus suppressing intracellular survival of *M.tb* (85). These are the cumulative host defense strategy for controlling *M.tb* infection. Nevertheless, *M.tb* has evolved to surpass the host defense strategy by targeting ER for deriving lipids and cholesterol for its own replication. Also, *M.tb* secretes CDP-diglyceride hydrolase (CdhM) that accumulates in ER, and damages its integrity, which induces cell death. This mechanism aids in *M.tb* dissemination to extracellular environment (86). Similarly, other *M.tb* proteins such as Rv0297 has PGRS domain, that enables the accumulation of CdhM in ER, and causes ER-induced apoptosis (87). The PE- PGRS domain containing proteins are antigenic in nature and are known for *M.tb* persistence during latent TB (88), hence are one of the potent targets for vaccine development.

It is remarkable to understand that ER stress results in halting in mRNA translation. This may ultimately ensue the accumulation of untranslated mRNA and other folded/unfolded proteins in a membrane less structure called stress granules (89). These stress granules are known to prevent ER mediated apoptosis in acute liver failure (90). This unexplored area of research in TB could serve as the another host defense strategy in preventing *M.tb* dissemination and thus controlling *M.tb* replication. This is important to realize that ER stress-induced apoptosis confers *M.tb* survival in later stages of granuloma and promotes dissemination (82). *M.tb* can counter host defense response at multitude level, hence it is noteworthy that researchers must dive in designing multi-epitopes vaccine (91). The multi epitope vaccines can target *M.tb* proteins that induce cellular stress and may target different arms of immune escape. For instance, our group has designed a peptide based multi-epitope vaccine (peptide-TLR agonist-liposome; PTL) harboring ESAT-6 and Ag85B. The intranasal delivery of PTL in conjunction with BCG, significantly improved the immune responses against murine model of TB (86). Despite numerous peptide- based multi-epitope vaccine in research pipeline against TB, limited research is available on the effects of these vaccines in combating cellular stress.

Nonetheless, this is a very less explored area of research to discover the role of ER stress during latency and reactivation. ER stress is potentially utilized by *M.tb* to persist within the host and evade from host killing mechanisms. Therefore, delineating the interaction between ER responses and *M.tb* is crucial in terms of designing better pharmacological therapies to limit *M.tb* progression.

### 3.3 Host microbiome

The various organs in human are co-inhabited by a diverse symbiotic microbial community. The majority of these microbiome reside in the respiratory tract and the gastrointestinal tract (92). The microbiota niche performs diverse protective functions in the host such as regulation of metabolism, maintaining barrier integrity, immune activation, trophic functions, etc. throughout the body, thereby maintaining tissue homeostasis (93). Several respiratory diseases including TB is characterized with changes in host microbiome composition in lung and gut which dictates the course of the disease pathogenesis.

#### 3.3.1 The respiratory system microbiome

The upper respiratory tract acts as a gateway to the lower respiratory tract and to the final destination of *M.tb* colonization- the lung (94). Similarly, metabolites produced by the gut microbiome can reach the lungs through blood (95). Several studies have hypothesized that dysbiosis of the microbiota in the upper respiratory tract, lungs, and gut increases the chances of *M.tb* primary infection, reinfection, and reactivation of LTBI (96).

Microbiome colonization is initiated post birth in the mucosal sites. In healthy human lungs, resident microbiome belongs to the following phyla- *Firmicutes*, *Bacteroidetes*, *Actinobacteria*, and *Proteobacteria*. Among these, the most prevalent genera have been identified as following- *Prevotella*, *Streptococcus*, *Veillonella*, *Fusobacterium*, and *Haemophilus* (97).

Recent literature suggests an alteration in the microbial community of the upper respiratory tract upon infection with TB. The microbial composition is speculated to have a role in the disease progression from healthy to ATB or LTBI. ATB shows enhancement in gram-positive and gram-negative species. *Proteobacteria, Bacteroidata*, and *Bacillota* are the most observed phylum while *Balutia* and *Acinoetbacter* are the dominant classes in ATB as compared to the Healthy cohort (HC). HCs were observed to have enrichment in the *Lacnospiraceae* family and the *Faecalibacterium* genus. Comparative analysis between the HC and LTBI cohort signified the presence of the *Kosakonia* genus in HC while the *Faecalibacterium* genus was enriched in LTBI (98).

Prospective cohort studies have indicated the role of the microbial community in predicting the susceptibility to TB. Notably, Healthy Household Contacts (HHCs) who did not acquire LTBI had an elevated relative abundance of *Corynebacterium, Dolosigranulum, Cutibacterium*, and *Staphylococcus*. Conversely, in HHCs with established LTBI, the abundance of *Corynebacterium, Lawsonella, Paracoccus, Cutibacterium, Dolosigranulum*, and *Moraxella* was notably higher. It is worth mentioning that the bacterial genera *Dolosigranulum* and *Variovax* were exclusively found in HC, leading to the conclusion that these genera are integral components of a healthy nasopharyngeal microbiome. Furthermore, in pre-LTBI patients, the absence of *Dolosigranulum*, coupled with the presence of *Rothia* and *Megasphaera* genera, has raised speculation regarding their potential involvement in modulating host immunity and increasing susceptibility to *M.tb* infection (99).

In a separate investigation led by Botero et al., noteworthy alterations were observed in the bacterial and fungal communities within the oropharynx of TB patients when compared to individuals in good health. The study unveiled a significant increase in the presence of the *Ascomycota* phylum in TB patients, indicative of fungal diversity shifts. Conversely, the genus *Cryptococcus* showed a notable decline in abundance within the oropharynx of TB patients. It is crucial to acknowledge that one of the key limitations of this study is the relatively small sample size, which may impact the generalizability of the findings (100).

These findings suggested a resemblance in nasopharyngeal species diversity and composition among HC, ATB, and LTBI groups, shedding light on the intricate interplay between upper respiratory microbiota and TB infection. These studies have however failed to conclusively show an association between bacterial dysbiosis in the nasopharyngeal tract and the chances of acquiring TB or progressing to LTBI.

Most of the studies focussing on studying lung microbiome diversity in TB rely on sputum samples for their downstream analysis (101). Sputum samples are spuriously contaminated with pharyngeal microbiota; thus, the result fails to be a true indicator of the lung microbiome (102). The investigation of the accurate picture of the lung microbiome is hindered by invasive techniques such as bronchioalveolar lavage fluid (BALF) collection and throat swabs (102). These techniques provide a better picture of the lung microbiome than the traditional sputum collection method.

In the pursuit of a deeper understanding of the lung microbiome, Xiao et al., conducted a study that harnessed BALF samples and employed metagenomics to explore microbial enrichment at the species level. Their findings revealed intriguing disparities between the HC and the Untreated Pulmonary TB Group (UTG). Specifically, the HC displayed enrichment in species like *Prevotella melaninogenica* and *Ralstonia pickettii*, while the UTG exhibited a heightened presence of *S. aureus* and *Neisseria gonorrhoeae.* These outcomes starkly contrast with those reported by Hu et al., in their BALF-based investigation (103).

Another pertinent study conducted by Pérez et al., shed light on differences in alveolar microbiota among patients with pulmonary TB. Their analysis of BALF samples uncovered a reduction in the *Streptococcus* genus, while concurrently identifying an increase in the abundance of *Mycobacterium, Lactobacillus*, and *Acinetobacter* (104). These findings underscore the complexity and variability in lung microbiota profiles associated with pulmonary TB, emphasizing the need for further research to elucidate the underlying factors and implications of these differences. These two studies provide crucial insights into the lung microbiome in TB patients but are severely limited by the patient sample size.

In a comprehensive study by Kang et al., the investigation unearthed distinct characteristics within the airway microbiota that set patients with *M.tb* infection apart from those without. Notably, a novel genus, *Anoxybacillus*, exhibited an increased presence in BALF samples. What sets this study apart is its robust patient sample size, which bolsters the need for further exploration to establish a definitive correlation between the prevalence of *Anoxybacillus* and TB (105).

Similarly, Zhou et al., research delivered significant findings, underscoring the prevalence of the *Cupriavidus* genus as dominant in TB patients, while *Streptococcus* emerged as the dominant genus in healthy subjects. Notably, their study was carried out utilizing BALF samples (106). These observations underscore the utility of BALF in uncovering crucial distinctions in lung microbiota between TB patients and healthy individuals, thus emphasizing the potential significance of these findings in understanding TB pathogenesis and treatment strategies. Despite the vast literature available, there are limited studies linking microbiome alteration to functional disease progression.

Xia et al., undertook a study with the objective of establishing a connection between changes in the microbial composition and variations in cytokine levels within BALF and their potential implications in the progression of TB (107). This investigation aimed to elucidate the interplay between microbial alterations and cytokine dynamics, shedding light on their role in the advancement of TB.

Meta-analysis serves as a valuable avenue for reinforcing scientific findings when the available sample size is constrained. These comprehensive studies amalgamate data derived from multiple investigations centered on a common research question, effectively surmounting the limitations posed by small sample sizes. Notably, a meta-analysis conducted by Hong et al., offered intriguing insights by proposing a co-abundance relationship between *R. mucilaginosa* and *A. graevenitzii* with *M.tb* within the lung microbiota of patients with ATB. It is important to acknowledge that this study is bounded by the number of source studies employed and the origin of the microbiota samples used to mimic the lung microbiome. Nonetheless, it introduces the intriguing possibility that the co-occurrence of specific bacterial communities may serve as a valuable indicator of disease progression and hold potential for predicting treatment outcomes (108).

#### 3.3.2 Gut microbiome

Microbiome in healthy gut harbor around 50 different bacterial phyla with *Bacteroidetes* and the *Firmicutes* as the two dominant gut resident phyla (109). TB progression is marked by dysbiosis of resident gut microbiome (110). Several cohort studies undertaken on the Chinese population indicate differences in diversity, composition, and function of gut microbiota in HC vs TB patients. The studies suggested dysbiosis of the gut microbiome in TB patients with a lower abundance of short-chain fatty acid producers namely microbiome genera such as *Lachnospiraceae* and species - *Haemophilus parainfluenzae, Roseburia inulinivorans*, and *Roseburia hominis*. TB patients also show a notable increase in opportunistic pathogens of the genera *Enterococcus* and *Rothia*. The studies suggest dysregulated host metabolism such as decrease in the biosynthesis capacity of amino acids and fatty acids and altered carbohydrate metabolism patterns in the gut in TB patients as compared to HC (13). Butyrate, a short-chain fatty acid, is a key metabolic pathway that is downregulated in notably all the cohort studies involving active TB patients (111). Butyrate production is inversely linked with the severity of inflammatory and metabolic diseases (112).

Demographics, geographic characteristics, and antibiotic usage also impact microbial diversity. Cohort studies from Pakistani, Chinese and Indian populations show differential enrichment of gut microbial phyla in healthy and TB patients (113).

In the Chinese cohort phylum *Actinobacteria* increased while phylum *Bacteroidetes* diminished in healthy cohort while in TB patients *Proteobacteria and Bacteroidates* have been notablyenriched. *Firmicutes* which shows enrichment in the Pakistani cohort significantly diminished in the Chinese cohort. While Firmicutes and Actinobacteria showed relative enhancement in a cohort study in the Indian population (13).

These microbial signatures need further investigation as the majority of the study uses TB and HC which does not provide confirmatory results on how these signatures might differ from non-TB inflammatory diseases (113).

#### 3.3.3 Gut-Lung axis

The secondary metabolites, immune signals and microbial remnant produced by gut/lung microbiome can affect the microbiota composition and abundance in a bidirectional fashion termed as the gut lung axis. The resident microbiome composition can dictate the disease progression. Recent murine studies have demonstrated that enriching the murine gut with *Lactobacillus* can alleviate asthma-like symptoms in mice (114).

Moreover, administration of IPA produced by gut *Clostidia* species have shown anti-tubercular activity in murine model by targeting trp and Short chain fatty acids (SCFA) biosynthesis in *M.tb* (115). It is worth noting that patients with ATB often exhibit increased regulatory T cells (Tregs). Interestingly, Faecal transplantation in murine model have shown to bear fruitful results in reducing T reg population and increasing protective cytokines such as IFN-γ/TNF-α (116).

**Figure 2:**
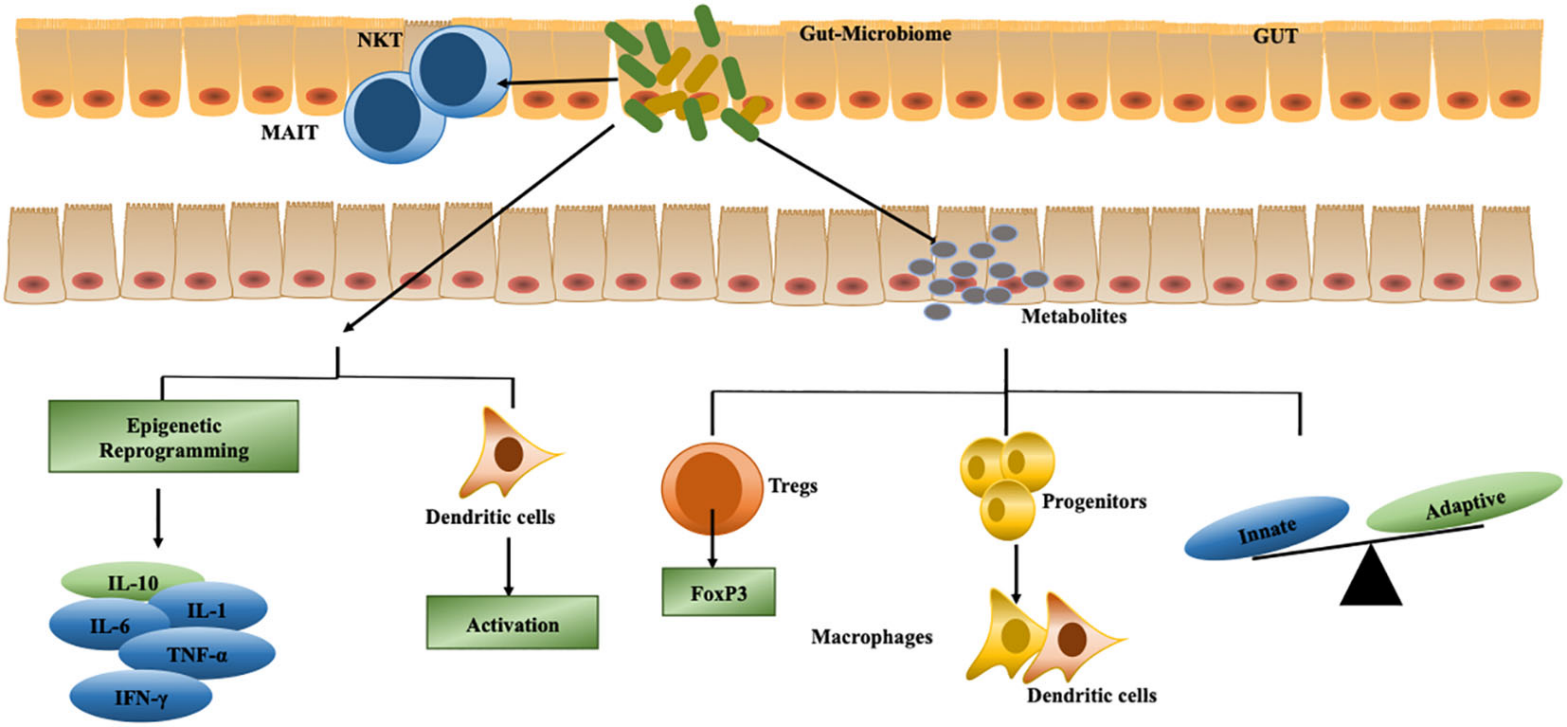
Microbiome controls various host cellular processes to regulate *M.tb* survival. Gut microbiota secretes various metabolites such as short-chain fatty acids (SCFA), indole propionic acid (IPA), flagellin, lipopolysaccharide (LPS), and peptidoglycan to regulate differentiation of immune cells that traverse to the lung for containing *M.tb* replication. These metabolites also regulate the balance of innate and adaptive immunity. Apart from protecting against TB, a few metabolites secreted by gut microbiota suppress immune responses against *M.tb* by enhancing Tregs function in the lung. Also, the microbiome in the respiratory tract can reprogram immune cells to secrete differential cytokines and induce dendritic cell activation to enhance antigen presentation to T cells.

SCFA produced by gut microbiome can enter the circulatory pathway and reach various peripheral organs including bone marrow. Within the bone marrow SCFA can induce differentiation of progenitor macrophage and DC which can then migrate to the lung and thus modulate lung microenvironment (117) (**Figure 2**). Anaerobic microbiota in the lower airways produce SCFAs, and elevated serum SCFA levels have been linked to the induction of FoxP3-expressing Tregs in BALF lymphocytes. Pulmonary SCFA produced by anaerobic microbiota has also been associated with a decrease in IFN-γ levels in active TB (118, 119).

Metabolites from the gut commensal microbiome have role in steering the abundance and function of non-conventional T cells such as Natural killer T cells (NKT) and Mucosal associated invariant T cells (MAIT). Studies using germ-free mice have shown the complete absence of MAIT cells, thus pointing towards a probable role of microbiota in affecting the abundance of MAIT cells. MAIT cells have been found to play important role in protection against TB (96).

Although there is limited research on the modulation of the gut-lung axis in TB, an intriguing study conducted by Negi et al., in a murine TB model highlights the role of dendritic cell activation by the gut microbiome of TB. This research suggests gut dysbiosis can lead to low mincle expression on lung DC which negatively regulates antimycobacterial T cell activation in the lung. The study suggests restoration of *Lactobacillus* as a possible measure to augment mincle based DC signaling (120).

Inflammation mediated by IFN-γ expression is crucial for controlling the initial stages of TB progression. Shen et al., revealed possible association of lncRNA-CGB produced by gut *Bacteroides fragilis* in positively regulating IFN-γ production by epigenetic reprogramming (121). The long-term anti TB treatment is known to induce significant alteration in microbial community. The altered microbiome can impact effective immune responses generation during the treatment (122). Limited literature is available that can explain the mechanism of lung microbiome mediated changes in the gut microbiome composition upon the initiation of colonization of lung by *M.tb*.

#### 3.3.4 Role of microbiome in modulating vaccine efficacy

The mechanism linked to generation of optimal vaccine responses in human is a very complex phenomenon. Several intrinsic and extrinsic factors such as age, genetics, nutritional status, pre-existing immunity and comorbidities dictate the nature of generation of vaccine responses (123). Recent studies have demonstrated yet another intrinsic factor of microbiome that can influence vaccine immunogenicity (124). Antibiotics-induced gut microbiome dysbiosis has been linked to diminished immune response generation to vaccination in healthy adults (125). Optimal vaccine-induced antibody response has been linked to the prevalence of gut commensals in diseases such as bacterial diarrhea, influenza, polio, etc (126). Studies using germ free animal models have linked defective Th1/Th2 responses, reduced intraepithelial lymphocytes, reduced antibody generation and a dysregulation in Th17 cell responses to the host microbiome (127).

In the context of TB, study conducted by Nadeem et al., reported gut microbiome dysbiosis as important determinant in dictating BCG vaccine efficacy in TB. The altered microbial community had a decreased prevalence of *Bifidobacterium, Lactobacillus* and an increased prevalence of *Bacteroides*. The dysbiosis resulted in decreased activation and frequency of various T cells subsets including the memory subset. The dysbiosis also impacted the secretion of pro-inflammatory cytokines and overall hampered the successful clearance of *M.tb* in the murine model of TB (128).

A cohort study of western European population suggested the role of gut microbiome in altering BCG-induced specific and trained immune responses. The trained immune response against *Staphylococcus aureus* was negatively correlated with the abundance of *Roseburia*. *Roseburia* secreted metabolites affected the phenylalanine metabolism. The disturbed phenylalanine metabolism can dampen BCG-induced trained immunity. The abundance of *Ruminococcus* and *Eggerthella lenta* was correlated to be a positive indicator for *M.tb* control (129).

Actinobacterial abundance was correlated with positive T-cell response to BCG administration in a cohort study on Bangladeshi infants. The prevalence of *Enterobacteriales*, *Pseudomonadales*, and *Clostridiales* was demonstrated to be associated with neutrophilia and reduced vaccine efficacy (130).

Immunomodulatory molecules produced by microbiota such as flagellin, peptidoglycan, lipopolysaccharides, and SCFA acts as ligands to PRRs (Toll like receptors, NOD-2 receptors) and modulate innate and adaptive immune cells activation and metabolism. SCFA production has been linked to the generation of efficient B cell response through modulating B cell differentiation into antibody producing cells (131). Gut microbiome has also been shown to regulate antigen specific T cell responses. The microbiota has notable role in generation of type-1 interferon by plasmacytoid dendritic cells which can eventually signal conventional dendritic cells to prime T cell more efficiently (132). Microbial-encoded cross-reactive epitopes could also potentially modulate vaccine immune response (124). Administration of bacterial species in form of pro/prebiotics, or faecal transplant can be an important determinant towards optimizing microbiota composition. This optimization can prove to be beneficial therapeutic avenue to positively impact vaccine immunogenicity (133) Thus, understanding and harnessing the influence of the gut microbiome on vaccination responses could lead to more effective and targeted strategies for disease prevention.

### 3.4 Exosomes

Exosomes are extracellular vesicles in the size range of 30-150nm. Exosomes are secreted from cells by the process of exocytosis and mediate cellular signaling and communication. Exosomes present surface protein from the parental cell as well the target cell. This feature can help in predicting the dynamic cellular turnover and organ specific metastasis of cellular contents, thus overcoming the limitation of conventional blood-based biomarker. The content of exosomes is highly diverse consisting of soluble and membrane bound proteins, lipids, and nucleic acids (134). Overall, the exosomes can be an excellent blood-based biomarker for early disease diagnosis and predicting disease progression. Blood-based biomarkers have the potential to significantly enhance TB detection, particularly in cases involving children and elderly individuals who may not be able to produce sputum (135). This approach also mitigates the risk of TB transmission to healthcare workers during sputum collection. In the pursuit of point-of-care diagnostics, exosomes offer a promising platform for blood-based TB detection. Additionally, exosomes can serve as an excellent vaccine development and delivery system (136).

Exosomes are an important component in the cellular signaling cascade. Extracellular vesicles (EVs) derived from *M.tb*-infected cells are commonly found to express *M.tb* cell wall, cell membrane, respiration proteins and lipoproteins (137). A report from a paediatric cohort followed by a validation study in Non-Human Primate (NHP) model suggests differential expression of *M.tb* cell wall components in ATB vs LTBI cases. Downregulation of Lipoarabinomanan (LAM) and upregulation of lipoprotein LprG have been observed in NHPs with latent infection. ATB results in upregulation of LAM and LprG in serum EVs of NHP (138). Multiple reaction monitoring assays have identified *M.tb* secretory antigens such as Culture filtrate protein (CFP2) and Mtp32 peptides as potential biomarkers for active TB, while in individuals with LTBI, the SVF peptide from Glutamine synthetase (GlnA1) have been detected in approximately 82% of participants (139, 140).

Exosomes in the bloodstream carry small RNAs, including microRNAs (miRNAs), which are well-protected from nucleases and are under active investigation as essential blood-based biomarkers, particularly in cancer. In a pilot study, miR-484, miR-425, and miR-96 were found to be significantly upregulated in patients with active TB compared to HC. These altered miRNAs have functional relevance in metabolic disorders and insulin signaling, which is reflected in disrupted sugar metabolism in macrophages infected with *M.tb* (141).

Profiling miRNAs from serum exosomes in individuals with LTBI and ATB, in comparison to HC, revealed differential expression patterns among the groups. Specifically, LTBI patients exhibited unique miRNA profiles, including hsa-let-7e-5p, hsa-let-7d-5p, hsa-miR-450a-5p, and hsa-miR-140-5p. In contrast, ATB exhibited an enrichment of hsa-miR-1246, hsa-miR-2110, hsa-miR-370-3p, hsa-miR-28-3p, and hsa-miR-193b-5p. Several other miRNAs associated with the activation of phagocytosis (has-mir-142-3p), apoptosis (has-mir-29a), and inflammatory signaling pathways were upregulated in TB patients (142). In another report, ATB has been documented to be characterized by the enhanced expression of let-7i-5p, miR-148a-3p, miR-21-5p, and miR-423-5p. In contrast, LTBI has been associated with upregulation in the expression of miR-122-5p and miR-451a (143). Studies characterizing miRNA from patient serum exosomes are very limited and vary extensively between different cohorts. The studies also lack functional in-depth characterization of miRNA targets and downstream signaling events that might prove to be beneficial or detrimental to the host. Several factors such as host sex, age, basal immunity, pre-existing disease, exosome isolation protocols, etc contribute to variation in exosome characteristics and functions (144). These confounders need to be explored in-depth to utilize exosomes as a highly precise diagnostic tool.

In addition to *M.tb* peptides, exosomes also transport host peptides with altered regulation. Comparative proteomic studies using isobaric tags for relative and absolute quantification (iTRAQ) revealed deregulated host proteins in serum extracellular vesicles when comparing ATB, non-TB (respiratory diseases other than TB; NTB), and HC. Host proteins such as Kynurenine-oxoglutarate transaminase 3 (KYAT3), Alpha-1-Antitrypsin (SERPINA1), Haptoglobin (HP), and Apolipoprotein C-III (APOC3) are primarily involved in neutrophil degranulation, plasma heme scavenging, kynurenine metabolism, and lipid metabolism, respectively (145) and shown to exhibit deregulation in ATB. It is essential to note that while there have been numerous studies characterizing the content of exosomes in the context of TB, most of them have focused on *in vitro* and *in vivo* models. Only a limited number of studies have been validated in large patient populations and the functional implication of the altered metabolism in TB progression Additionally, many studies have primarily compared ATB to HC, neglecting the consideration of NTB and LTBI.

Furthermore, the response to TB treatment in DOTS responders versus non-responders has not been extensively explored and validated in the context of differences in exosomal content. An open randomized controlled trial comparison of patient plasma exosome proteins suggested downregulation of Complement component binding protein 4 (C4BPA) and Calgranulin B (S100A9) might be involved in a positive host response to LTBI treatment. S100A9 which is a calcium binding protein, has been reported to be upregulated in chronic pulmonary obstructive disease and notably impairs innate immune cell polarization in obesity models (146, 147). C4BPA has a functional role in modulating host-*M.tb* interaction (148). Exosomes are being speculated to have a role in the development and pathogenesis of drug resistance phenotypes. Drug-resistant TB (DR-TB) has a dysregulated cholesterol metabolism in comparison to non-drug-resistant TB (NDR) patients. DR-TB patients show a decrease in the expression of various apolipoprotein family members such as APOA1, APOB, and APOC1. These apolipoproteins have been linked to the formation of foamy macrophages and reduction of cellular uptake of anti-TB drugs thus leading to failure of DR-TB treatment (149). Several pieces of research are being conducted to evaluate urine-based *M.tb* diagnostic assays which can be used as adjunct detection methods for smear and PCR-negative patients (150, 151).

One of the important limitations of blood-based circulating biomarkers is that they provide inconclusive detail about immune processes taking place in human tissues. Emerging studies suggest liquid biopsies can provide an alternative to the standard circulating biomarkers assays. Liquid biopsies are currently being used for prenatal testing, cancer monitoring, predicting vaccine efficacy, and investigating cases of graft rejection (152).

Liquid biopsies are been widely researched in the quest to improve the diagnosis of TB. Cf nucleic acids are primarily released by dying cells. Profiling of CfDNA coupled to epigenetic marks on cfDNA can provide information about mechanisms of cellular injury, death, systemic immune dynamics, and interaction of immune cells in tissues (152, 153). cfDNA in humans can have host as well as microbial origin. In a proof-of-concept study using CRISPR-Cas13, mycobacterial regions of IS6110 and IS1081 were detected in three separate active TB patient cohorts. Future studies can help elucidate the correlation of microbial cfDNA distribution in different states of TB progression (154). Monitoring DNA methylome in immune cell-derived cfDNA can prove to be of paramount importance in understanding immune responses and cellular exhaustion in inflammatory and chronic diseases (152).

#### 3.4.1 Role of exosome in vaccine and immunotherapy

In addition to the role as biomarkers, exosomes have the potential to be used for drug delivery systems due to their nano-lipid properties (155). Several *in-vitro* and *in-vivo* studies demonstrate the immune-activating properties of *M.tb*-infected innate immune cell-derived exosomes. *M.tb* antigen-loaded exosomes have demonstrated immune boosting properties which are critical in vaccine and adjuvant development for developing novel vaccine and immunomodulatory candidates (156, 157).

Exosomes carrying potent *M.tb* antigens hold a great promise to serve as a productive cell-free vaccine candidate to replace/boost BCG immunizations. Exosomes from *M.tb*-infected macrophages can direct the polarization of naïve macrophages. Additionally, exosomes derived from infected innate immune cells can effectively prime the adaptive T cells in the *in-vivo* model of TB (16). In yet another study on the murine model of TB, exosome vaccination has been found to prime as well as boost BCG immunization. Mice displayed a Th1-mediated immune response with limited Th2 response as compared to BCG-immunized mice. The purified exosomes used for vaccination showed notable enrichment with antigenic mycobacterial proteins (157).

EV derived from human neutrophils stimulated with *M.tb* results in a decrease in intracellular *M.tb* in the macrophages. The EVs derived from the *in-vitro* stimulated human neutrophils contains TLR ligands and co-stimulatory molecules required for innate and adaptive immune cell activation, respectively. The EVs induce autophagy to clear intracellular *M.tb* from the macrophages (158). Another research suggests the use of ubiquitin-tagged Ag85B-ESAT6 fusion protein-based exosome can be used as a novel vaccine candidate. This antigen-specific exosome-based delivery system has been observed to induce enhanced IFN-γ response by T cells and it enhances mycobacterial clearance (159).

Exosomes have also been tested as vaccine candidates for cancer in murine melanoma models. DC-derived exosomes were found to induce adaptive immune responses. The exosomes significantly augmented CD8^+^ T cell proliferation and differentiation into central memory cells (160). EVs have also been found to act as a superior adjuvant and elicit an effective cell-mediated immune response (161). In a study conducted on the murine model, exosomal adjuvant enhanced the IFN-γ secretion upon immunization with hepatitis B surface antigen. The role of exosomes as adjuvants during BCG immunization requires further research in TB (162). Exosomes function as a double-edged sword in the host-pathogen interaction. There are few studies suggesting exosomal contents can limit inflammation and delay mycobacterial clearance (16, 163).

Nevertheless, more research in the exciting field of exosomes is required to realize its full potential as a vaccine and adjuvant candidate to provide better protection against TB.

## 4 Concluding remarks

*M.tb* has developed diverse defensive strategies to counter host immune responses, targeting nearly every significant host process. Importantly, disruptions in any host cellular process intricately influence connected pathways, thereby regulating *M.tb* pathogenesis. For instance, *M.tb* causes a shift in metabolic pathways through impacting immunometabolism that can induce ER stress. The Unfolded protein-initiated ER stress leads to the inclusion of unfolded proteins, spliced mRNA, and metabolites within EV. The EVs secreted out of the infected cells alter immune signaling in the adjacent bystander immune cells. The immune mediators and the intermediatory metabolic pathway products can also affect the diversity and composition of host microbiome in the various organs. Cohort studies suggests the dysregulation of gut/respiratory microbiome is correlated to *M.tb* pathogenesis. The metabolites generated by host and the host harbored microbiome can modulate signaling events that affect *M.tb* growth, survival and interactions within the host. This is noteworthy that dysregulation of the microbiome also affects the metabolism of immune cells which impacts the energy status of the cell, and consequently alters immune responses against *M.tb*. Through this review, we have highlighted the less explored host pathways that prevail in *M.tb* pathogenesis. Currently, many researchers are investigating the effect of these integrated pathways in *M.tb* pathogenesis. Yet, extensive efforts are needed to understand the modulations in infected cells or activated immune cells that collectively affect *M.tb* pathogenesis. Despite having many vaccines and drug candidates in clinical pipelines, a well-designed approach to prevent TB and reduce tissue damage is still an urgent need to provide better protection against TB. In conclusion, it is necessary to design novel host directed therapies that can improve the protection against TB.

## Author contributions

AG: Data curation, Investigation, Writing – original draft, Writing – review & editing. AV: Data curation, Investigation, Writing – original draft, Writing – review & editing. AB: Data curation, Formal Analysis, Investigation, Visualization, Writing – original draft, Writing – review & editing. VD: Conceptualization, Funding acquisition, Investigation, Resources, Supervision, Visualization, Writing – original draft, Writing – review & editing.
